# The Mechanisms Involved in Morphine Addiction: An Overview

**DOI:** 10.3390/ijms20174302

**Published:** 2019-09-03

**Authors:** Joanna Listos, Małgorzata Łupina, Sylwia Talarek, Antonina Mazur, Jolanta Orzelska-Górka, Jolanta Kotlińska

**Affiliations:** Department of Pharmacology and Pharmacodynamics, Medical University of Lublin, Chodzki 4a St., 20–093 Lublin, Poland (M.Ł.) (S.T.) (A.M.) (J.O.-G.) (J.K.)

**Keywords:** opioid receptors, adenylate cyclase activity, morphine tolerance and withdrawal signs, mesolimbic system, mitogen-activated kinases (MAP kinases), β-arrestin

## Abstract

Opioid use disorder is classified as a chronic recurrent disease of the central nervous system (CNS) which leads to personality disorders, co-morbidities and premature death. It develops as a result of long-term administration of various abused substances, along with morphine. The pharmacological action of morphine is associated with its stimulation of opioid receptors. Opioid receptors are a group of G protein-coupled receptors and activation of these receptors by ligands induces significant molecular changes inside the cell, such as an inhibition of adenylate cyclase activity, activation of potassium channels and reductions of calcium conductance. Recent data indicate that other signalling pathways also may be involved in morphine activity. Among these are phospholipase C, mitogen-activated kinases (MAP kinases) or β-arrestin. The present review focuses on major mechanisms which currently are considered as essential in morphine activity and dependence and may be important for further studies.

## 1. Introduction

Among opioid drugs, morphine, codeine, fentanyl or buprenorphine are considered the most effective analgesics for application in post-operative and cancer pain. Their chronic administration is associated with a high rate of abuse potential [1]. Moreover, other opioids, including heroin, are used as recreational drugs and have the ability to induce opioid dependence. Substance use disorder is classified as a chronic recurrent disease of the central nervous system (CNS) which leads to personality disorders, co-morbidities and premature death [2,3]. Substance use disorder develops as a result of long-term administration of substances with abuse potential and include physical dependence and/or psychological addiction. Physical dependence is associated with the formation of neuroadaptive changes in the CNS, both at the molecular and cellular level [4,5,6,7,8,9,10,11]. These changes are responsible for the occurrence of characteristic withdrawal signs after cessation of drug-taking. The type and severity of withdrawal signs depend on various factors, such as the type of abused drug, drug doses, time period of drug use, patient’s age, age of the first use of drugs or genetic predispositions [12,13]. Psychological dependence is defined as compulsive drug use to improve the perception of well-being [14]. A typical syndrome of psychological addiction in humans includes intensified drug-seeking behaviour, compromised capacity of will, compulsive drug intake despite awareness of its harmful effects, as well as persistent and recurrent obsession, even after years of abstinence.

Scientific research shows that despite numerous social, psychological and medical projects aimed at reducing the phenomenon of substance abuse, the number of individuals with opioid use disorders is steadily increasing worldwide. Nowadays, opioid dependence is considered a global public health crisis. According to the World Health Organization, opioid overdose deaths increased from 69,000 people in 2014 [15] to 118,000 in 2015 [16]. Dramatic increases in maternal opioid use and neonatal abstinence syndrome also has been observed during the last decade. Therefore, in response to the opioid crisis, scientific and government efforts should be focused on several priorities: improving pain management with non-dependent drugs; promoting knowledge of opioid risks; or providing support for cutting-edge research on pain and addiction. The aim of this review is to present current knowledge on mechanisms involved in morphine activity which develop after its acute or chronic administration. The understanding of morphine mechanisms is important for further studies on the activity of the opioidergic system.

## 2. Morphine and Its Receptors

Morphine and other opioid drugs are able to induce a broad spectrum of pharmacological activity. Occurring in the CNS, they induce strong analgesia, euphoria, sedation, endocrine dysregulation, miosis, antitussive activity or respiratory depression. Additionally, they induce muscle spasms and histamine release in the peripheral nervous system. Observed in clinical practices, there are many opioid drugs that mainly are used as analgesics (Table 1).

The pharmacological action of acute doses of morphine is associated with stimulation of the opioid receptors. It interacts predominantly with the μ opioid receptors. Generally, opioid receptors can be divided into subtypes: μ (μ1, μ2, μ3); δ (δ1, δ2, δ3); and κ (κ1, κ2, κ3) [13,62]. The novel nociception/orphanin FQ receptor is considered to be a non-opioid branch of the opioid receptor family [13]. Opioid receptors are a group of G protein-coupled receptors [63]. They consist of seven transmembrane domains, three extracellular and three intracellular loops, extracellular amino acid *N*-terminus and intracellular carboxyl C-terminus. Opioid receptors are located both in the central and peripheral nervous system. The first data on localisation of opioid receptors in the nervous system appeared in 1973 [64]. Nowadays, it is known that opioid receptor subtypes are located in areas involved in: 1) pain transmission, such as the thalamus, rostroventral medulla (RVM), periaqueductal grey area (PAG), pons or in the spinal cord of the dorsal horn; 2) the rewarding system, such as the nucleus accumbens, ventral tegmental area or the cortex; 3) other brain areas, such as the hypothalamus, amygdala, ventral pallidum, globus pallidus, nucleus raphe, hippocampus and olfactory bulb [64,65,66,67]. They also occur in peripheral tissues, for example, in the gastrointestinal and in the respiratory tract [67,68]. 

The localization of opioid receptors in the gut [69] is responsible for regulation of gastrointestinal motility and secretion [70]. Consequently, μ-opioid receptor agonists inhibit gastric emptying, increase pyloric muscle tone, and delay transit through the small and large intestine. All these effects lead to constipation—one of the most impactful adverse effects of morphine and other opioid drugs.

The presence of opioid receptors in the respiratory tract is associated with important clinical indications of morphine [71]. Morphine is used as an antitussive drug, also after surgery within the respiratory system, and in the control of pain due to lung cancer. Conversely, the overdose of morphine induces a high risk of respiratory depression, which is an important limiting factor in morphine therapy [72].

Endogenously, opioid receptors are stimulated by endogenous peptides, such as endomorphins, dynorphins and enkephalins. Endomorphins consist of four aminoacids, including two endogenous ligands (endomorphin-1 and endomorphin-2) that have the highest affinity and selectivity for the µ-opioid receptor in the central and peripheral nervous systems. They are involved in analgesia and reward. Dynorphins (dynorphin A and dynorphin B) exert their effects primarily through the κ-opioid receptor and have less affinity for the μ-opioid receptor and δ-opioid receptor. Enkephalins (met-enkephalin and leu-enkephalin) produce the effect mainly on δ receptors, but they also have an affinity for μ receptors.

## 3. Molecular Effects of Acute and Chronic Dose of Morphine

A binding of an endogenous (endomorphin molecule) or exogenous (morphine molecule) ligand with an opioid receptor leads to activation of a Go or Gi protein and to subsequent phosphorylation by a family of kinases called the G protein-coupled receptor kinases (GRKs). This induces molecular changes inside the cell, including β-arrestin binding. G protein is composed of three subunits: α, ß and γ. Binding of the ligand to the receptor results in opioid receptor activation by GTP binding to the α subunit, while the α-GTP complex dissociates from the dimer ßγ-subunits. Both complexes, α-GTP and dimer ßγ, participate in intracellular signal transduction. This leads to an inhibition of adenylate cyclase activity and a reduction of cyclic adenosine monophosphate (cAMP) levels in the cell [73,74], as well as suppression of the activity of protein kinase A [75,76]. α-GTP also activates phospholipase-C (PLC) and mitogen-activated protein (MAP) kinases pathways [77]. PLC hydrolyses phosphatidylinositol 4,5-bisphosphate (PIP2) into inositol 1,4,5-trisphosphate (IP3) and diacylglycerol (DAG). IP3 increases calcium release from the endoplasmic reticulum that activates calcium-dependent signalling. The activation of potassium channels (G-protein gated inward rectifying potassium channel–GIRK-3) is also observed [78], leading to increased hyperpolarisation of the cell, and, indirectly, to reduced cell excitability [77]. The ßγ dimer directly blocks the calcium channel (P/Q-type, *N*-type, and *L*-type channel) and reduces calcium concentration [79] in the cell, leading to suppression of other neurotransmitters. The effect of stimulation of opioid receptors on the activity of potassium and calcium channels was repeatedly confirmed in various brain areas (hippocampus, nucleus locus coeruleus, the area abdominal caps, etc.), and this mechanism has been considered a key effect for the stimulation of the opioid receptors [80,81,82,83].

Chronic exposure to morphine induces the phosphorylation of opioid receptors by GRKs. This phosphorylation prepares opioid receptors for arrestin binding. Arrestin binding blocks further G protein-mediated signalling, thereby, inducing desensitization of opioid receptors [84].

**Figure 1:**
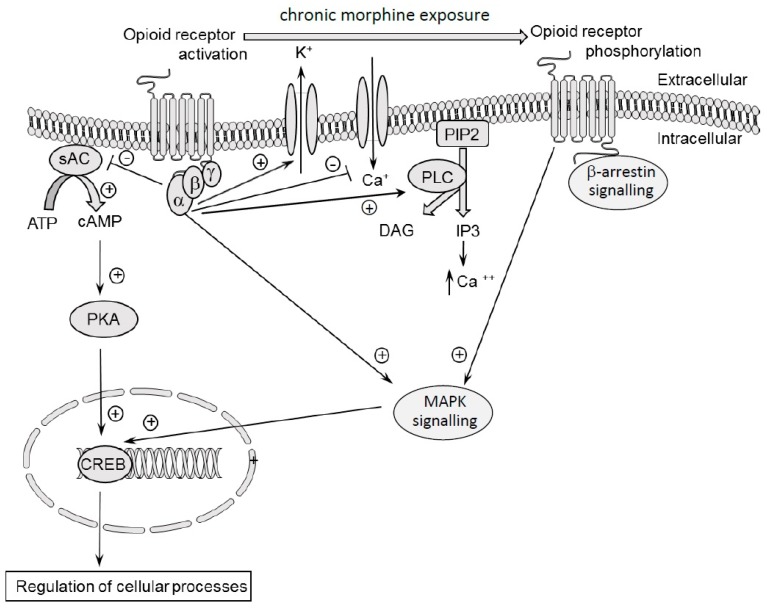
Molecular mechanisms of morphine action. A binding of ligand with an opioid receptor activates Go or Gi protein. G protein is composed of three subunits: α, β and γ. The ligand binding results in opioid receptor activation by GTP binding to the α subunit. The α-GTP complex dissociates from the dimer βγ-subunits. Both complexes: α-GTP and dimer βγ, participate in intracellular signal transduction. This leads to an inhibition of adenylate cyclase activity and reduction of cAMP level and protein kinase A inside the cell. The activation of potassium channel and cellular hyperpolarisation is observed. The βγ dimer blocks the calcium channel and reduces calcium concentration inside the cells. The chronic stimulation of opioid receptors by morphine induces the phosphorylation of opioid receptors. sAC–soluble adenylyl cyclase; PKA–protein kinase A; CREB–cAMP response element binding protein; PIP2–phosphatidylinositol biphosphate; PLC–phospholipase C; DAG–diacylglycerol; IP3–inositol triphosphate; MAPK–mitogen-activated protein kinases.

Thus, clinically important pharmacological effects of morphine, induced by a single administration of this substance, are connected with multidirectional, molecular mechanisms which occur within the cell. The molecular effects of morphine are shown graphically in Figure 1.

## 4. Opioid Analgesia

Opioid analgesia is associated strongly with activation of the μ opioid receptors located in CNS. These receptors are localized in subcortical regions of the brain, as previously mentioned, from which the descending pain pathways originate, such as in the thalamus, the PAG and the RVM, as well as in the spinal cord’s dorsal horn [85]. Occurring at the supraspinal level, opioid analgesics stimulate the μ opioid receptors located on GABAergic interneurons in the RVM, hence, decrease GABA release. Physiologically, GABA, by acting on GABA-A receptors, suppresses the “OFF” cells in the RVM, which subsequently raises the action potential. When the GABA level is reduced, the tonic inhibition of “OFF” cells is relieved (i.e., disinhibition) and the “OFF” cells’ signal suppresses pain perception in the spinal cord (descending pain regulation). Additionally, opioid-induced activation of μ opioid receptors on GABAergic “ON” cells in the RVM inhibits the firing of these cells. Thus, the disinhibition of “OFF” cells and the direct inhibition of “ON” cells produce analgesia, an effect which can be measured using thermal nociception tests [86]. Additionally, the amygdala, a brain area responsible for emotional states, indirectly can modify pain transmission.

Regarding the spinal level, opioid-induced analgesic effects are mediated by the activation of presynaptic μ opioid receptors localized in the dorsal horn of the spinal cord. The triggering of these presynaptic receptors causes membrane hyperpolarization. Such changes in membrane polarization lead to the inhibition of mediators of the pain pathway, such as glutamate, substantia P and calcitonin gene-related peptide (CGRP) from nociceptive primary afferent neuron terminals. Consequently, the ascending pain pathway transmission is attenuated.

**Figure 2:**
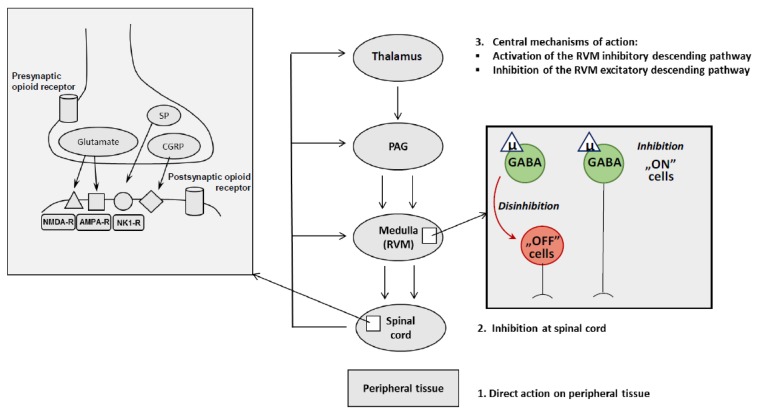
Mechanisms of morphine analgesia. Regarding the supraspinal level, opioid analgesics stimulate the μ receptors located on GABAergic interneurons in the RVM decreasing GABA release. GABA suppresses the “OFF” cells in the RVM, which subsequently raises the action potential. Additionally, opioid-induced activation of μ opioid receptors on GABAergic “ON” cells in the RVM inhibits the firing of these cells. Observed at the spinal level, opioid-induced analgesia is mediated by the activation of presynaptic μ opioid receptors localized in the dorsal horn of the spinal cord. PAG–periaqueductal gray in midbrain; RVM–rostral ventromedial medulla; GABA–gamma-aminobutyric acid; SP–substance P; CGRP–calcitonin gene-related peptide; NMDA-R–N-methyl-D-aspartate receptor; NK1-R–neurokinin-1 receptor; AMPA-R–α-amino-3-hydroxy-5-methylisoxazole-4-propionic acid receptor.

It should be noted that opioid induced analgesia is a complex process in which μ opioid receptors can be heteromerized with δ or κ opioid receptors. Heterodimeric associations between μ–δ opioid receptors, for example, can be used as a model for the development of novel combination therapies for the treatment of chronic pain and other pathologies [87]. The mechanisms of opioid analgesia are shown graphically in Figure 2.

## 5. Opioid Rewarding Effects

Generally, the rewarding effect of various addictive substances, including opioids, is associated with stimulation of structures within the mesolimbic system, such as the ventral tegmental area and the nucleus accumbens. This increases the dopamine release in the nucleus accumbens [88] which determines the feeling of pleasure. However, the impulses from other brain structures, such as the ventral striatum, hippocampus, prefrontal cortex or amygdala, also may stimulate the mesolimbic system [8], affecting dopamine levels in the nucleus accumbens. Thus, a dramatic escalation in drug intake, with extended access to drug self-administration, is characterized by a dysregulation of rewarding dopamine pathways in the brain [89,90,91]. Therefore, the rewarding effect of morphine and other opioids is associated with stimulation of μ opioid receptors localized at the GABAergic terminals of the ventral tegmental area. Such stimulation inhibits GABA release that, in turn, disinhibits dopaminergic neurons and leads to the release of dopamine in the nucleus accumbens that induces feelings of euphoria and promotes the development of drug dependence [13,92,93]. 

**Figure 3:**
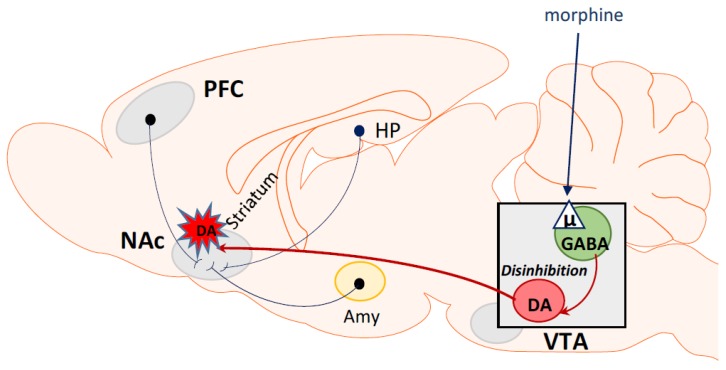
Mechanisms of morphine-induced rewarding effect. The rewarding effect of morphine is associated with stimulation of μ opioid receptors localized at the GABAergic terminals in VTA. It inhibits GABA release and disinhibits dopaminergic neurons in NAc. PFC (prefrontal cortex); NAc (nucleus accumbens); HP (hypothalamus); Amy (amygdala); VTA (ventral tegmental area); GABA (gamma–aminobutyric acid); DA (dopamine).

While dopamine plays a crucial role in the rewarding action of morphine, many neurotransmitters and neuromodulators in the CNS affect the dopaminergic system and indirectly modulate various aspects of morphine addiction. These neurotransmitters include glutamate [94,95], serotonin [96], γ-aminobutyric acid (GABA) [97,98], noradrenaline [96,99], adenosine [91], nitric oxide [100], orexin [101], and others. Pharmacological manipulation of these neurotransmitters found in the reward pathway potentially can modify craving for drugs of abuse. The mechanisms of a morphine-induced rewarding effect are shown graphically in Figure 3.

## 6. Morphine as a Dependent Drug

Chronic morphine abuse leads to physical and psychological dependence [8,12,14,102]. Physical dependence on morphine is manifested by characteristic withdrawal symptoms that can develop after abrupt cessation of drug administration [103,104]. Morphine withdrawal symptoms in people include sneezing, runny nose, cough, abdominal pain, diarrhoea, anorexia, anxiety and other effects [105,106]. Withdrawal effects observed in animals include jumping, paw tremors, teeth chattering, wet dog shakes and diarrhoea [103,105,107,108]. Morphine withdrawal symptoms are evoked in experimental studies either by discontinuation of chronic morphine administration or via the administration of opioid receptor antagonists. Naloxone, the most commonly used opioid receptor antagonist in experimental pharmacology, usually is applied in a dose range from 1 to 6 mg/kg [109,110,111]. The severity of morphine withdrawal symptoms is analyzed on the basis of the number of withdrawal episodes [103,111] (Table 2). 

Some authors suggest that the decrease in dopamine concentrations in the mesocorticolimbic system plays a critical role in morphine withdrawal [112,113,114,115]. Still, neurotransmitters such as noradrenaline [116], glutamate [117], serotonin [118], orexin [119] and cortisol [120] also may be involved in morphine withdrawal. Furthermore, these changes in neurotransmitters are accompanied by changes in cell signalling pathways such as a significant increase in cAMP level [121] and the deregulation of the MAP kinase pathway (ERK 1/2) [122,123].

Morphine tolerance, the second parameter of physical dependence on this substance, is defined as a need to increase the morphine dose (a rightward shift in the dose–response curve) to achieve the same pharmacological effect [124]. The phenomenon of tolerance develops regarding analgesic, euphoric, sedative, respiratory depressant, and nauseating effects of opioids, but not to their effects on miosis and bowel motility (constipation) [125]. Generally, there are three types of tolerance: pharmacokinetic tolerance; learned tolerance; and pharmacodynamic tolerance. Pharmacokinetic tolerance refers to changes in the distribution or metabolism of the drug, while learned tolerance refers to a reduction in the effects of a drug due to compensatory mechanisms that are learned, such as behaving normally while still intoxicated. The most important form of tolerance relevant to opioids is pharmacodynamic tolerance. This type of tolerance has been related to neuroadaptive changes that take place after long-term exposure to the drug, including changes in receptor density and alteration in receptor coupling to G proteins and signal transduction pathways [126]. 

During experimental studies, morphine tolerance commonly is analyzed by means of nociception, measured in behavioural tests, like the tail immersion test [103,127] or the hot plate test [128,129] (Table 2). Morphine tolerance may be modified by various neurotransmitters including dopamine [130,131], but also serotonin [132], acetylcholine [133], orexin [134] or endocannabinoids [135] and others. 

Behavioral sensitization is a phenomenon involving escalating behavioral responses to repeated exposure to a stimulus such as a drug of abuse like cocaine or opioids, after a drug-free period which can be long-lasting—even to the extent of many years. This effect is related closely to the environment in which the addictive substance is taken. Behavioural sensitization is an important parameter in evaluating the degree of psychological addiction. Animal studies reflect the drug-seeking behaviour in people which often leads to drug use relapse [14]. Experimentally, behavioural sensitization is commonly manifested and measured as an increase in locomotor activity of animals after administration of a challenging dose of the abused substance [136,137]. Furthermore, behavioural sensitization also may be expressed as the enhanced rewarding effect of the addictive substance. During animal studies, this can be observed in the conditioned place preference test [100,138,139]. Less commonly, sensitization may be observed through the intensification of withdrawal signs after repeated withdrawal periods [91,140] (Table 2).

The development of behavioural sensitization occurs through the neuroadaptive changes observed in glutamatergic and dopaminergic neurotransmission [102,141,142,143]. Sensitization is associated with an increased dopamine release in the mesolimbic structures [14,144,145]. The main pathways involved in behavioural sensitization are the dopaminergic pathway from the ventral tegmental area and the glutamatergic pathways from the prefrontal cortex, both terminating in the nucleus accumbens. 

The expression of morphine sensitization is associated primarily with an increased dopamine release and with alterations in the sensitivity of dopaminergic D1 receptors in the mesolimbic structures, including the striatum, nucleus accumbens, ventral tegmental area, hippocampus and the prefrontal cortex [4,5,6,7,91,146,147]. Observed in rats, the pharmacological blockade of D1 receptors impairs the expression of sensitization [148]. Similarly, the administration of D1 and D2 receptor antagonists into the nucleus accumbens also inhibits the development of behavioural sensitization in rats [147]. Moreover, published data show that the increased expression of D1 receptors in the shell of the nucleus accumbens, observed during the morphine sensitization process, is associated with elevated MAP kinase activity (ERK 1/2) and this effect is reduced by the D1 receptor antagonist—SCH 23390 [149]. Alternatively, the development of behavioural sensitization is associated more with the glutamatergic system and with the ventral tegmental area because the antagonists of NMDA and AMPA receptors inhibit the acquisition, but not the expression, of behavioural sensitization [150,151]. Thus, various neuroadaptive changes, including alterations in the density of receptors, the neurotransmitter level or deregulation in cell signalling, can be responsible for the expression and acquisition of behavioural sensitization.

## 7. Molecular Mechanisms of Morphine Tolerance and Dependence 

Considering the cellular level, the major effect of an acute dose of morphine is the decrease in cAMP level and hyperpolarization that is induced by changes in the activity of potassium and calcium channels. However, chronic stimulation of opioid receptors by morphine and other opioid ligands induces adaptive changes within opioid receptors. This leads to a decrease in the acute receptor response. Such changes are essential in controlling the receptor activity as they protect receptors against hyperstimulation, promote signal termination and regulate their expression [86]. Consequently, desensitization, internalization, resensitization or downregulation of opioid receptors is developed. These mechanisms lead to the attenuation of the pharmacological activity of morphine and other opioid drugs, which often is observed after chronic exposure to them. 

Regarding chronic morphine exposure, it was previously suggested that the down-regulation of µ receptors was responsible mainly for the reduced morphine activity which was manifested as tolerance. This hypothesis, however, was not confirmed in experiments because chronic exposure to morphine did not produce down-regulation of μ receptors [152,153]. Similarly, internalization of μ receptors was previously considered as a neural mechanism underlying morphine tolerance. However, in vitro studies do not confirm the hypothesis because morphine is able to induce a strong tolerance but its effect on μ receptor internalization is poor [154]. Nowadays, an increasing number of evidences link mechanisms of tolerance with desensitization of μ opioid receptors. It should be underlined that the definitions of cellular tolerance and desensitization are similar and, for many years, these terms have been confused. Both are defined as the reduced capacity to respond to the same drug dose. However, desensitization (expressed as “rapid tolerance” in earlier studies) means a progressive agonist-induced reduction of signal transduction in opioid receptors seen during in vitro models, while tolerance is observed during in vivo models. Desensitization develops directly after opioid exposure and is reversed rapidly in agonist-free circumstances [84]. Rapid desensitization depends on potassium and calcium ion activity, while sustained desensitization is related to enzyme activity (adenylyl cyclase or MAP kinases). Nowadays, the enhanced desensitization of opioid receptors is considered as being an important mechanism of morphine tolerance, which results from numerous neuroadaptive changes. The desensitization may be caused by increased adenylate cyclase activity and an elevated cAMP level, for example, which, in turn, affects the activity of the cAMP response element-binding protein (CREB) [155]. Additionally, the desensitization involves G protein uncoupling because, in morphine treated animals, the binding of the GTPα complex is reduced in comparison with control animals. Moreover, morphine induced desensitization of µ receptors is associated closely with deregulation of β-arrestin-1 and β-arrestin-2 levels in the cell [156,157,158]. β-arrestin, a cytosolic protein, is bound to the opioid receptor surface after opioid receptor phosphorylation by a class of serine/threonine kinases (GRKs). Activation of β-arrestin inhibits further cell signalling, which directly produces the receptor desensitization [86,159]. The process of desensitization also is produced by an increase in phosphorylation of MAP kinases. MAP kinases have a large range of potential substrates, including transcription factors controlling gene expression. The role of extracellular signal-regulated kinase 1/2 (ERK1/2) in the effect of chronic morphine administration also was confirmed, although these results are contradictory. Narita et al. [160] and Macey et al. [161] observed that chronic exposure to morphine induced the increase in ERK1/2 phosphorylation, while other authors showed the lack of effect [162,163,164]. There also are some data showing the role of phospholipase C in morphine-induced desensitization [165]. Phospholipase C increases the level of other secondary neurotransmitters, such as inositol– (1,4,5) –triphosphate (IP3) and 1,2-diacylglycerol (DAG). These lead to the elevation of calcium levels in the cell. The phospholipase C also catalyzes the release of arachidonic acid from cell membranes participating in the formation of inflammation [166]. Phospholipase C plays a significant role in morphine activity. The inhibitors of phospholipase C potentiate the antinociceptive effect of a single dose of morphine and reduce morphine tolerance [167,168].

The molecular effects of morphine tolerance and dependence is demonstrated graphically in Figure 1.

## 8. Epigenetic Mechanisms of Morphine Tolerance and Dependence

Novel data shows that chronic exposure to abused drugs may induce complex epigenetic interactions within a genome thereby regulating patterns of gene expression [169,170,171]. These epigenetic modifications include changes in DNA methylation [172], histone acetylation and demethylation, alterations in DNA accessibility, and chromatin structure modification. They are inherited despite a lack of effect on DNA structure. First evidence on the role of DNA methylation and histone deacetylation in µ opioid receptor expression was published in 2007 [173]. Observed in P19 mouse embryonal carcinoma cells, hypermethylation of DNA silences µ opioid genes at the transcriptional level and DNA demethylation induces higher µ opioid gene expression. Moreover, µ opioid receptor expression also was increased after pharmacological manipulation, such as administration of a demethylating agent (5′-aza-2′-deoxycytidine) and histone deacetylase inhibitors. It was demonstrated in another study that chromatin modification also participates in µ opioid gene expression [174]. Mashayekhi et al. [175] documented that the alternations in mRNA levels of brain-derivative neurotrophic factor, BDNF, in the ventral tegmental area and the locus coeruleus of rats on the seventh day of morphine abstinence were associated with histone modifications. Another study also confirmed the role of histone methylation in the effects of chronic morphine exposure [176]. Ciccarelli et al. [177] found the augmentation of histone acetylation during naloxone-precipitated morphine withdrawal in the shell of the nucleus accumbens and in the lateral septum. There also are experiments showing that pharmacological inhibition of DNA methylation by 5′-aza-2′-deoxycytidine have an influence on morphine place preference in rats [178,179]. Recent study confirmed the existence of epigenetic changes in numerous brain structures (among others: cerebral cortex, cerebellum, hippocampus, hypothalamus, medulla oblongata, etc.) after acute and chronic exposure to opiates [180]. 

Although much progress has been made in understanding the mechanisms of opioid activity, little is known about the mechanisms of transcriptional regulation. It seems they are essential regulators of gene expression. Further studies are necessary to define the precise links between the epigenetic alterations and behavioral effects of morphine and other opioids.

## 9. Biased Opioid Ligands as a New Class of Opioid Analgesics

Since traditional morphine-like drugs hold many adverse effects (itching, constipation, nausea/vomiting, respiratory depression or abuse lability), recently, the ligand biased at the G protein-coupled receptor has been synthesized. These compounds were perceived to preferentially stimulate certain intracellular pathways over others and produce less side-effects. Thus, such drugs could be safer, more effective and well-tolerated then morphine [181]. Thus far, several G protein-biased μ opioid receptor (GPB–MOR) agonists have been developed [182]. They preferentially activate Gαi protein signalling connected with analgesia over β-arrestin signalling that mediate some undesirable effects. TRV130 (oliceridine), was the first such agonist progressed to clinical trial [183]. Another GPB–MOR ligand is a compound referred to as PZM21, which represents the first example of the structure-based discovery of a biased G protein-coupled receptor ligand. PZM21 showed initial promise in animal studies as a potent analgesic without respiratory depression and morphine-like reinforcing effects [184]. However, although GPB–MOR may produce less respiratory depression and gastrointestinal dysfunction at analgesic doses than currently available opioid analgesics, they retain their abuse liability [185]. Other promising therapeutic candidates for pain and itch relief are G protein-biased agonists for the κ opioid receptor [186]. The example of such a compound is Triazole. It did not alter locomotor activity and did not cause sedation in animal models. These effects appeared to arise from its inability to decrease the dopamine release in mouse striatum, thus not adversely affecting the dopaminergic transmission. Furthermore, this compound did not influence the reward circuit in the brain, thus did not trigger signs of dysphoria and aversion, unlike a typical κ opioid agonist. Generally, the discoveries of new pathways and new ligands at the μ/ κ opioid receptor highlight the opportunities for biased ligands as a new class of analgesics providing more efficacious and safer relief from moderate-to-severe pain.

Summing up, morphine, acting on opioid receptors, induces a broad spectrum of pharmacological activity. However, long-term morphine administration generates dysregulation at cellular and molecular levels in the brain, leading to addiction. Despite extensive studies, the effective management of opioid disorders is limited. Therefore, the recognition of mechanisms underlying morphine/opioid dependence seems to be extremely important in searching for new strategies of therapy for morphine/opioid abusers. The present review summarizes the current knowledge on morphine activity and provides a major overview of the mechanisms involved in its acute and chronic exposure.

## Figures and Tables

**Table 1:** Agonists of µ receptors which are useful in clinical practices.

Drug Names	Structural Formula	Indications
**Alfentanil**	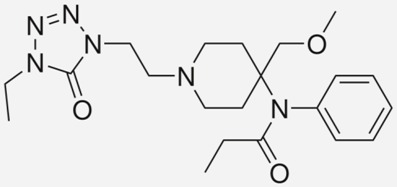	- anaesthesia in surgery [17]
**Buprenorphine**	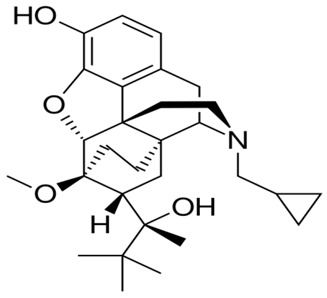	- relieve moderate-to-severe pain [18] - substitute treatment for opioid addiction [19] - Neonatal Abstinence Syndrome [20]
**Butorphanol**	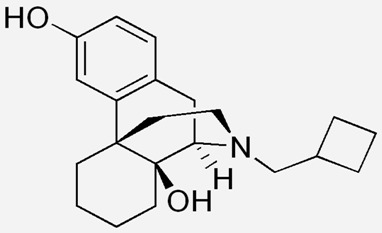	- treat moderate-to-severe pain [21] - relieve acute morphine-induced pruritus [22]
**Codeine**	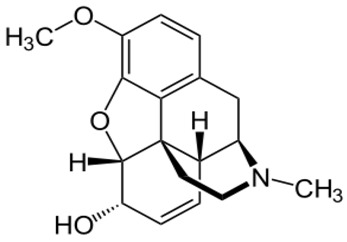	- treatment of chronic cough [23] - relief of moderate-to-severe pain [24] - treat persistent diarrhoea [25]
**Dextromethorphan**	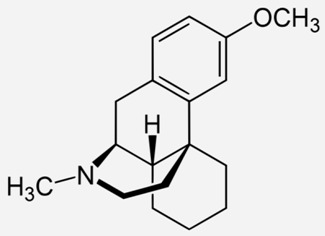	- temporary relief of coughs without phlegm [26]
**Diphenoxylate**	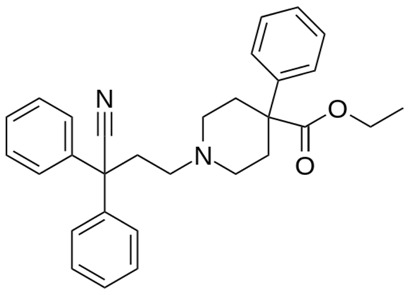	- acute and chronic diarrhoea of various origins [27] - reduction in the amount of faecal fluid after ileostomy and colostomy [28]
**Dihydrocodeine**	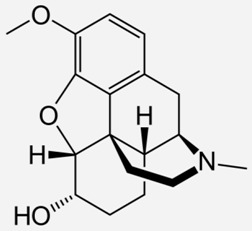	- treat moderate-to-severe pain [29] - treat dry cough [29] - treat diarrhoea [29]
**Fentanyl**	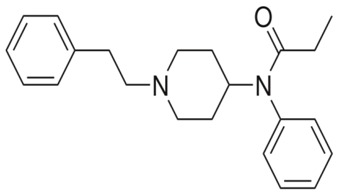	- treatment of severe, chronic pain [30] - used for surgical anaesthesia [30]
**Hydrocodone**	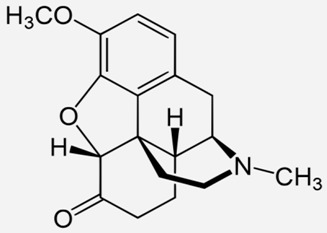	- the management of pain severe enough to require daily, around-the-clock use [31]
**Hydromorphone**	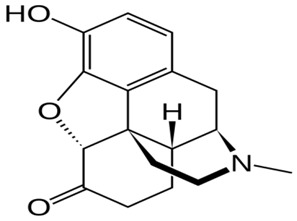	- relieve moderate-to-severe pain [32]
**Laevodropropizine**	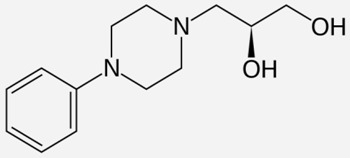	- treat dry cough [33]
**Levorphanol**	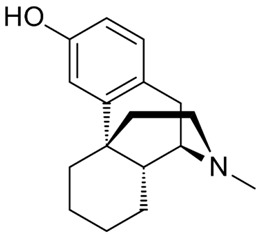	- use in moderate-to-severe pain [34]
**Loperamide**	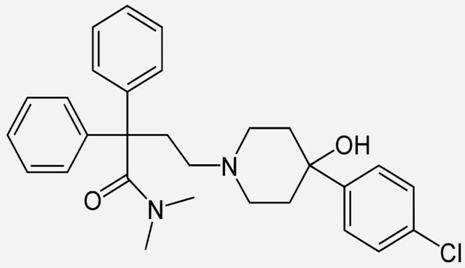	- stop diarrhoea [35]
**Meptazinol**	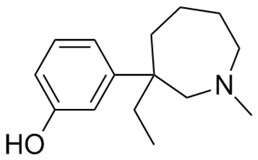	- relieve moderate-to-severe pain (among others, obstetrics) [36]
**Methadone**	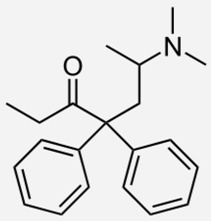	- treatment of opiate dependence [37] - the treatment chronic, severe pain [38] - treatment of neonatal abstinence syndrome [39]
**Morphine**	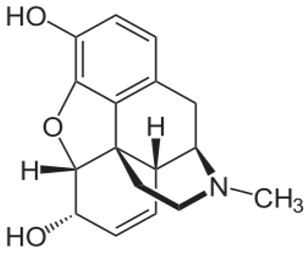	- moderate-to-severe pain relief [40] - used for procedural sedation [40] - sporadically used as an antitussive drug [41] - treatment of Neonatal Abstinence Syndrome [39]
**Nalbuphine**	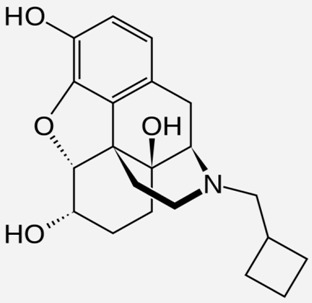	- itching treatment [42] - recommended for weak-to-moderately severe pain [42] - sedation [43] - anaesthesia [44]
**Nalmefene**	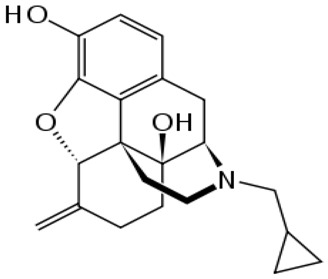	- reduction of alcohol consumption [45]
**Naloxone**	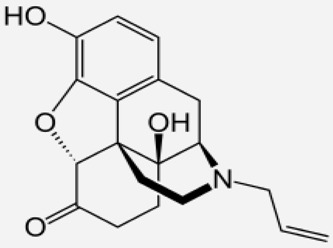	- Treatment of poisoning, overdose of opioid substances [46] - reversal of undesirable effects from opioid used during anaesthesia [47] - counteracting the occurrence of opioid-induced constipation [48] - substitute treatment of opioid dependence [49]
**Naltrexone**	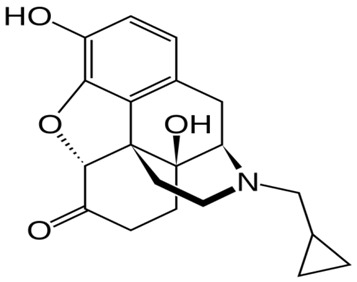	- treatment of alcoholism [50]
**Oxycodone**	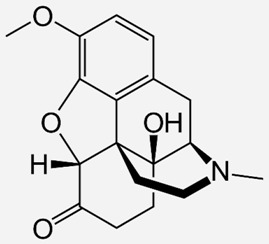	- treatment of opioid-induced constipation [48] - pain treatment [51]
**Pentazocine**	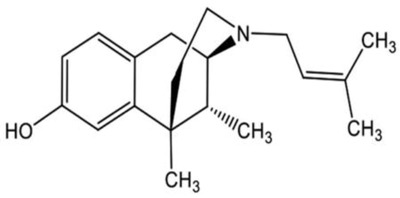	- treatment of moderate-to-severe pain [52] - a preanaesthetic or preoperative medication [52]
**Pethidine**	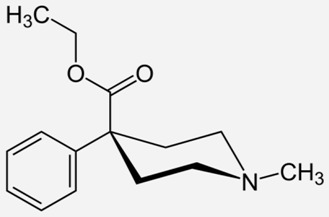	- the treatment of moderate-to-severe pain [53] - used as an adjunct to preoperative medications to reduce shivering [53] - relief of childbirth pain [54]
**Remifentanil**	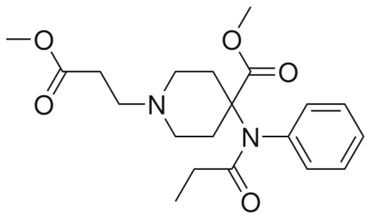	- relief of childbirth pain [54] - treatment of moderate-to-severe pain [55] - providing anaesthesia and sedation [55]
**Sulfentanil**	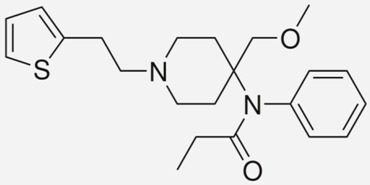	- management of moderate-to-severe pain [56] - anaesthesia [56]
**Tapentadol**	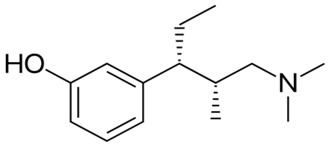	- to help relieve moderate-to-severe pain [57]
**Tilidine**	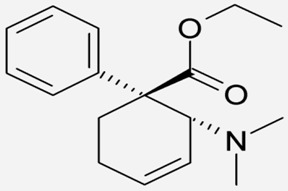	- treatment of pain [58]
**Tramadol**	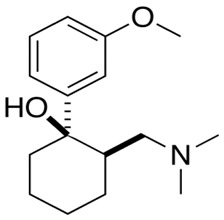	- treat moderate-to-severe chronic pain [59] - relief of childbirth pain [59] - anaesthesia [60] - premature ejaculation [61]

**Table 2:** The experimental procedures in particular phases of addiction.

Experimental Procedures for Phases of Addiction
Morphine dependence is obtained commonly by chronic administration of increasing doses (from 10 to 50–100 mg/kg) of morphine, twice a day for 5–9 consecutive days.
Morphine withdrawal is obtained in morphine dependent animals either by discontinuation of chronic morphine administration or via administration of an opioid receptor antagonist, such as naloxone, at a range of doses from 1 to 6 mg/kg. The severity of morphine withdrawal symptoms is analyzed on the basis of the number of withdrawal episodes, such as jumpings, paw tremors, teeth chattering, wet dog shakes and diarrhoea.
Morphine tolerance is obtained by repeated administration of the same dose of morphine (10 mg/kg) for several (3–7) consecutive days. Commonly, it is analyzed by comparison of the reaction of animals on nociceptive stimulus, recorded on the first and last day of morphine administration. Morphine tolerance commonly is measured in behavioural tests, such as the tail immersion test or the hot plate test.
Morphine-induced behavioral sensitization is related closely to the environment in which the addictive substance is taken and reflects morphine-seeking behavior in studied animals. It is obtained by administration of a challenge dose of morphine (at range of 1–10 mg/kg) in morphine dependent animals after several days (7–10) of a morphine-free period. It is measured as an increase in locomotor activity of animals, rarely as the enhanced rewarding effect.
